# The development and validation of a novel COVID19 stigma scale among healthcare workers (COVISS-HCWs)

**DOI:** 10.1186/s12913-022-08911-5

**Published:** 2022-12-05

**Authors:** Hasan Nabil Al Houri, Abdullah Alhouri, Rand T. Akasheh, Christine E. S. Jovanovic, Heba Al-tarcheh, Douaa Mohammad Nazir Arrouk, Ahmad Nabil Alhouri, Marah Marrawi, Malik E. Juweid, Youssef Latifeh

**Affiliations:** 1 Internal Medicine Department, Al Assad University Hospital, Al Mouwasat University Hospital, Damascus University, Damascus, Syria; 2 Department of Medicine, Division of Gastroenterology, Royal Berkshire Hospital, Reading, UK; 3 Department of Nutrition and Dietetics, American University of Madaba, Madaba, 11821 Jordan; 4 Department of Preventive Medicine, Northwestern University, Chicago, IL 60611 USA; 5 Department of Pulmonary Medicine, Al Assad University Hospital, Damascus University, Damascus, Syrian Arab Republic; 6 Applied Statistics – Quantitative Methods, Damascus University, Damascus, Syria; 7 Faculty of Medicine, Syrian Private University, Damascus, Syrian Arab Republic; 8 Department of Mathematical Statistics, Faculty of Science, Damascus, Syrian Arab Republic; 9 Department of Radiology and Nuclear Medicine, University of Jordan Hospital, Amman, Jordan; 10 Department of Psychiatry, Faculty of Medicine, Damascus University, Syrian Private University, Damascus, Syrian Arab Republic

**Keywords:** COVID-19, Stigma, Healthcare Workers, Validation, Novel, Scale

## Abstract

**Background and aim:**

Fear of coronavirus disease 2019 (COVID-19) and its complications may result in stigmatization of individuals who may carry the virus. This is of special concern to healthcare workers who tolerate additional physical and emotional stress at times of pandemic. The aims of this study are to (1) develop and validate the COVID-19 Stigma Scale (COVISS-HCWs) survey; and (2) investigate the experience of stigma among healthcare workers possibly dealing with COVID-19 patients in five major public hospitals in Damascus, Syria.

**Methods:**

We divided the sample into two parts and then underwent EFA on the first 350 participants, dividing the 14 questions into two dimensions. Furthermore, CFA was conducted on the other 350 participants to confirm how correctly a hypothesized model matched the factor structure by EFA, as described above. Moreover, the coefficient of determination (R2) and item–scale correlations (standardized factor loading) were estimated to establish the acceptability of the final structure of the COVISS-HCWs. Through a cross-sectional study, a convenience sample of 700 healthcare workers participated in a self-administered questionnaire containing a section for demographic variables and another for newly designed COVISS-HCWs. The scale comprises 14 adapted and novel items that measure two subscales: feelings of perceived harm and inferiority, and avoidance. Descriptive statistics, reliability, and validity were evaluated.

**Results:**

The 14 COVISS-HCWs items were reduced to 11 items with a high Cronbach’s α of 0.909. A significant correlation was observed between the responses to each COVISS-HCWs item and the corresponding subscale, and between each subscale and the overall scale. Feeling stigmatized was reported by 9.86% of the participants. Younger age, low socioeconomic status, and higher intensity of contact with COVID-19 patients significantly correlated with higher stigmatization.

**Conclusions:**

The novel COVISS-HCWs is a reliable and valid tool to evaluate stigma among healthcare workers during the COVID-19 pandemic. The Stigma prevalence among healthcare workers was 9.86%. Therefore, this must be addressed to prevent possible psychosocial and public health repercussions.

## Introduction

The coronavirus disease which emerged in the year 2019, (COVID-19) is caused by the novel severe acute respiratory syndrome coronavirus-2 (SARS-CoV-2) [1]. The disease has assumed pandemic proportions leading to the death of over 6 million people worldwide [1]. Limited knowledge about the disease and lack of effective means of control may result in feelings of fear and anxiety of catching the infection, its symptoms, and complications. Fear of the disease may lead to negative behaviors against persons who have the disease or may have it [2]. Public health strategies dealing with rapidly growing disease outbreaks such as COVID-19 require a delicate balance between maintaining precautionary measures for infection control such as physical distancing while alleviating the possible psychological harms of such measures [3].

The psychological repercussions of lockdowns and physical distancing policies have been well documented [4]. These, however, may be aggravated by the stigma of carrying the infection or being around those who have it. According to the World Health Organization (WHO), stigma is defined as “the negative association between a person or group of people who share certain characteristics and a specific disease” [5]. Stigma might be expressed verbally or through discriminatory behaviors such as isolation, refusal to provide service, bullying, and harassment. Stigma may also affect individuals dealing with those infected with the disease, such as caregivers, family members, or those in the same community. Importantly, healthcare providers may also face stigmatization. This, along with the well-documented high rates of anxiety and depression among this group, may compromise their well-being and hence, may even threaten disease control [6]. Studying stigma against patients with infectious diseases such as human immunodeficiency virus (HIV), and tuberculosis (Tb) is prevalent. However, studies regarding stigma against healthcare providers taking care of those patients are limited. In March 2020, the WHO Department of Mental Health and Substance Use developed a series of suggestions that can be used to support the psychosocial well-being of different groups during the outbreak [7]. According to Roy et al., stigma is associated with many health conditions. In contrast, awareness may minimize the stigma and facilitate acceptance of the healthcare workers in the general population [7]. To date, only two studies documented stigmatization using a specific questionnaire against healthcare workers dealing with COVID-19 patients [8, 9]. The aim of this study is to design and validate a stigma scale specific to COVID-19 and investigate correlates of stigma among healthcare workers dealing with COVID-19 patients in Syria. Through a novel questionnaire tailored to the COVID-19 context, we studied feelings of perceived harm, shyness and inferiority, and avoidance among healthcare workers in five major public hospitals in Damascus, the capital of Syria. The validity and reliability of this questionnaire are presented in this manuscript. In addition, we aimed to investigate whether the experience of stigma correlates with demographic or workplace variables and whether it relates to a healthcare worker’s attitude towards COVID-19 vaccines.

## Methods and materials

### Study design and setting

This is a cross sectional study conducted in five primary public hospitals (Al Assad University Hospital, Al Mouwasat University Hospital, Children’s University Hospital, Dermatology University Hospital and Obstetrics University Hospital) that work with patients during COVID-19 pandemic in Damascus, Syria. Data was collected between August 1^st^ and November 30^th^, 2020.

### Ethical considerations

This study was approved by the institutional review board (IRB) of Damascus University with a protocol approval number of (613). All methods were performed in accordance with the relevant guidelines and regulations. The Questionnaire contained detailed information about the study objectives. Informed consent was first obtained from participants who volunteered to fill the questionnaire. The anonymity and confidentiality of the data were ensured by assigning an identification number to each participant restricted to the research team.

### Sample selection

Healthcare workers (HCWs) were recruited through convenience sampling to participate in a self-administered questionnaire which was distributed by researchers via flyers. Respondents eligible for the study were adult male and female Syrian HCWs of at least 18 years of age. The sample included physicians, dentists, nurses, pharmacists, and other medical technicians who signed consent forms before data collection. In this study, the required number of participants ranged from 320 to 480 since the rule of thumb stated that 10–15 cases per candidate item are required. Therefore, the sample size of 700 participants included was adequate [10].

### COVID-19 stigma scale development

A self-reported questionnaire was developed to determine if healthcare workers experienced COVID-19-related stigma, regardless of whether they were in direct contact with COVID-19 patients or not. A scientific committee consisting of physicians including three psychiatrists reviewed the literature pertaining to disease stigma and extracted some of the survey questions from previously validated surveys for other diseases such as the HIV, and TB stigma questionnaires [11–16]. The questions were then amended to suit the context of COVID-19. Newly created questions by the study committee were also added to the survey.

### Measurement of demographics, stigma and correlates

The Questionnaire included three main parts: The first part consisted of eight questions regarding participant characteristics, including age in years, sex, place of residence, educational level, field of study, living status (flat, house, hostel), number of people living with the respondent, and financial status.

The second part of the questionnaire consisted of 14 questions Responses to each survey item was captured on a five-point Likert scale (1 = never, 2 = rarely, 3 = sometimes, 4 = very often, and 5 = always).

The third part of the questionnaire investigated aspects that may be related to stigmatization. The intensity of healthcare worker contact with COVID-19 patients was surveyed, and three responses were included: “no contact”; “only occasional contact for a few minutes with personal protective equipment (PPE)”; and “close daily contact with PPE”. Participants were also asked whether or not they had neighbors, relatives, or co-workers who were aware that they worked in hospitals or clinics, or that they were in contact with Coronavirus-infected individuals.

Moreover, the questionnaire investigated whether participants were willing to get a COVID-19 vaccine if it were to become available. The responses to this question included “I will not take it”; “I will take it without fear”; and “I will take it despite fear of its side effects”.

### Data collection

A detailed explanation of the study was presented to healthcare workers at participating hospitals and clinics. The researchers distributed paper questionnaires directly to healthcare workers. Each questionnaire had a cover letter explaining the study, its aims, and how to complete and return the form. Participants had to sign consent forms and the self-completed questionnaires were returned to the researchers directly.

### Statistical analysis

The Statistical Package for the Social Sciences (SPSS) software, version 23.0, was used for descriptive analysis. This included demographic characteristics of participants and frequency distributions of their responses to questionnaire items and to each subscale. The responses captured on a 1–5 scale were used to calculate means and standard deviations for each questionnaire item. Inferential statistics were conducted to test the correlations between stigma and different demographic variables. Chi-square test and post-hoc analysis using Tukey’s test where appropriate. The statistical significance level was set at (0.05).

### Questionnaire reliability

Internal consistency reliability of the COVID-19 stigma Scale was measured by Cronbach’s alpha and corrected item-total correlations. A Cronbach’s alpha of 0.80 or higher implied acceptable reliability.

### Questionnaire validity

A confirmatory factor analysis (CFA) was performed with maximum likelihood using AMOS version 23 data analysis software (Analysis of Moment Structures). Validation was conducted with a two-step approach was used. First: was dividing the sample into two parts then we underwent EFA on the first 350 participants. construct validity of the stigma scales was assessed we performed an EFA by a principal factor extraction method for the EFA cohort, with the factor obliquely rotated using the Promax criterion. The Kaiser-Meyer- Olkin measure and Bartlett test of sphericity were performed to ensure the appropriate use of factor analysis. Eigenvalues > 1.0 and the scree plot with the number of factors that explained > 5% of the variance were used to define the number of factors retained [17, 18]. To develop a practical and concise measurement tool we considered items acceptable if the loading coefficient was > 0.40.

The unidimensional set of items of the COVISS-HCWs was identified and underwent EFA which divided the 14 questions into two dimensions. The first dimension consisted of 10 questions while the second dimension consisted of 4 questions Table 1.

**Table 1:** Exploratory factor analysis of the 14-item stigma

Exploratory Factor Analysis of the 14-Item stigma (*n* = 350)				
Item		Factor Loadings^*^		Communality
		Factor 1	Factor 2	value
Q1	[You were subject to verbal abuse]	**.903**	-.316	.532
Q2	[Your personal freedom was restricted]	**.714**	-.078	.441
Q3	[People were curious to know about your patients’ COVID19 test results]	**.694**	-.103	.396
Q4	[You were blamed for the nature of your work and spreading the infection]	**.716**	.049	.563
Q5	[People seemed uncomfortable dealing with you]	**.577**	.276	.622
Q6	[You felt unwanted]	**.550**	.320	.642
Q7	[You worried about being stereotyped]	**.512**	.294	.550
Q8	[You have been stared or pointed at in your community]	**.459**	.309	.497
Q9	[You felt that others were scared from you]	**.627**	.207	.609
Q10	[You feared being negatively judged by others]	**.445**	.365	.549
Q11	[Your family or friends preferred not to keep their kids around you]	-.075	**.877**	.687
Q12	[People avoided eating or drinking with you]	-.104	**.918**	.726
Q13	[People kept a very large distance when interacting with you]	-.096	**.880**	.671
Q14	[People avoided any physical interaction with you]	-.052	**.848**	.662
Percentage of the variance		49.649	8.553	Total variance
				58.203

^*^The extraction method was principal component analysis, with the rotation method by oblique, promax rotation

Items load on the assigned factor loadings > 0.4 are highlighted

To test the two-factor structure model of COVISS-HCWs, A CFA was conducted on the other 350 participants to confirm how correctly a hypothesized model matched the factor structure by EFA, as described above. CFA was performed with maximum likelihood using AMOS version 23 data analysis software (Analysis of Moment Structures).

To determine the appropriateness of the tested model, we tested the fit indices, including the root mean square error of approximation, standardized root mean square residual, comparative fit index, and Tucker-Lewis Index. Moreover, the coefficient of determination (R2) and item–scale correlations (standardized factor loading) were estimated to establish the acceptability of the final structure of the COVISS-HCWs. The CFA in turn excluded three questions from the questionnaire as their R-Squared was less than 0.30 Table 2. Finally, the questionnaire consisted of two subscales that included 11 items: the first subscale is harmfulness and inferiority (7 Items) while the second one is avoidance (4 Items).

**Table 2:** The Composite Reliability (CR) and Average Variance Extracted (AVE), and Discriminant Validity Index Summary for all Constructs

The Composite Reliability (CR) and Average Variance Extracted (AVE), and Discriminant Validity In- dex Summary for all Constructs				
	CR > 0.70	AVE > 0.50	Feeling of Shyness and Inferiority Subscale	Avoidance Subscale
Feeling of Shyness and Inferiority Subscale	0.86	0.503	**0.71**	
Avoidance Subscale	0.84	0.593	0.659	**0.77**

Cronbach’s α coefficient was calculated to determine the internal consistency reliability. The Cronbach’s α coefficient of the overall scale was 0.909, exceeding our minimum acceptable value of 0.8, which indicates an excellent level of internal consistency. The Cronbach’s α for the 2 subscales of harmfulness and inferiority, and avoidance were 0.87 (very good), and 0.85 (very good), respectively.

Using modification indices, significant cross-loadings were incorporated. These cross-loadings were determined to be measuring the same construct and were co-linear, so the higher loading variable of the pairs was retained to arrive at the final model. Model fit was assessed using Akaike Information Criterion (AIC) and Bayesian Information Criterion (BIC), and convergent and divergent validity were evaluated.

## Results

### Characteristics of study participants

In analyzing the demographic characteristics of the study sample (Table 3), we found that most participants were between 18–29 years old (79.4%), females (61.6%), and living in Damascus (67.7%). Moreover, most were living in a family house (57%), in medium (38.6%) and good (46.6%) socioeconomic status and holding advanced degrees (62.9%). While most participants were working in internal medicine departments (44.7%), 20.6% were working in the nursing departments, and smaller percentages were working in other medical departments.

**Table 3:** Characteristics of study participants

**Variable (*N* = 700)**	**Categories**	N	%
Age	18–29	556	79.4
	30–49	114	16.3
	≥ 50	30	4.3
Gender	Female	431	61.6
	Male	269	38.4
Place of residence	Damascus	474	67.7
	Rif Dimashq	123	17.6
	Other	103	14.7
living condition	Flat (rent)	399	57.0
	University housing	144	20.6
	Family House	157	22.4
Including yourself, how many people live in your household?	Alone	283	40.4
	1, 2	155	22.1
	3, 4	220	31.4
	≥ 5	42	6.0
Financial status	Low	326	46.6
	Medium	32	4.6
	Good	270	38.6
	Excellent	72	10.3
Educational level	Diploma	417	59.6
	University degree	148	21.1
	Master’s degree	112	16.0
	Doctorate	23	3.3
Medical Specialty	Dentistry	16	2.3
	General surgery	38	5.4
	Internal medicine	313	44.7
	Nursing	144	20.6
	Obstetrics and gynecology	24	3.4
	Pediatric	14	2.0
	Pharmacy	40	5.7
	Special Medicine	58	8.3
	Special surgery	53	7.6
How extensive was your contact with people infected with COVID-19?	No contact at all	22	3.1
	Occasional, for a few minutes	273	53.3
	Daily, close contact with PPE	303	43.3

### Responses of healthcare workers to stigma question

Responses were captured on 1–5 Likert scale. The average response to each survey item was calculated to evaluate the most common responses. We found that the mean response of every item in all subscales was above 1.0 and less than 3.0. However, the spread in responses was relatively wide with standard deviations of mean ranging between 0.43—1.39 (Table 4).

**Table 4:** Reliability of COVID19 sigma scale

Survey Items	Factor loading (> 0.5)	Corrected item-total correlation (> 0.3)	Mean ± Standard Deviation
1) You were subject to verbal abuse	0.558	0.479	2.04 ± 0.99
2) Your personal freedom was restricted	0.522	0.509	1.88 ± 1.06
3) People were curious to know about your patients’ COVID19 tests	0.578	0.416	2.79 ± 1.39
4) You were blamed for the nature of your work and spreading the infection	0.610	0.660	2.30 ± 1.15
5) People seemed uncomfortable dealing with you	0.626	0.694	2.11 ± 1.00
6) You felt unwanted	0.630	0.717	1.75 ± 0.93
7) You worried about being stereotyped	0.628	0.705	1.84 ± 1.00
8) You have been stared or pointed at by people in your community	0.600	0.600	2.20 ± 1.11
9) You felt that others were scared from you	0.706	0.691	2.31 ± 1.09
10) You feared being negatively judged by others	0.576	0.637	1.95 ± 1.07
11) Your family or friends preferred not to keep their kids around you	0.737	0.632	1.77 ± 1.05
12) People avoided eating or drinking with you	0.790	0.664	1.60 ± 0.89
13) People kept a very large distance when interacting with you	0.642	0.602	2.11 ± 1.10
14) People avoided touching you	0.700	0.635	2.04 ± 1.09

Participants more frequently responded with “never” or “rarely” to the questionnaire items with percentages ranging between 6%—91.8% of the participants. Also, participants less frequently (0%—18.1%) responded with “very often” or “always”. This confirms that many participants reported experiencing some form of harmfulness and inferiority, and avoidance.

### Internal consistency reliability of COVID19 stigma scale

Table 7 presents our findings on COVID Stigma Scale reliability. The Cronbach’s α coefficient of the overall scale was 0.909, exceeding our minimum acceptable value of 0.8, which indicates an excellent level of internal consistency. The Cronbach’s α for the 2 subscales of harmfulness and inferiority, and avoidance were 0.87 (very good), and 0.85 (very good), respectively.

Moreover, we found a corrected item total correlation range of 0.214—0.717. With most items of the questionnaire meeting a minimum corrected item-total correlation of 0.3, and none of the items having negative correlations, this points to very good internal consistency.

### Validity of COVID19 stigma scale

To evaluate construct validity, Pearson’s correlation coefficients between the responses of each subscale and the responses of the whole stigma scale, and also between the two subscales were calculated. Correlation coefficients between the subscales (r = 0.651), and between the subscales and the overall scale (r = 0.858 – 0.949) were all significant (all P-values < 0.001), indicating that survey items work in harmony to measure COVID19 stigma with good level of validity (Table 5). Additionally, the responses to each item significantly correlated with its corresponding subscale (all P-values < 0.001). Pearson’s correlations ranged between 0.71—0.81 in the Harmfulness and inferiority, and 0.82- 0.85 in the avoidance subscale. Taken together, these data indicate that COVISS-HCWs items serve its purpose with good internal validity. Corrected item-total correlations of 0.30 were used as indicators of internal consistency reliability [19].

**Table 5:** Pearson’s correlations between the responses to each subscale and the overall scale

Pearson’s correlations between the responses to each subscale and the overall scale			
Construct	Harmfulness and Inferiority Subscale	Avoidance Subscale	COVISS (All Subscales)
Feeling of Shyness and Inferiority Subscale	1		
Avoidance Subscale	.651^**^	1	
COVISS (All Subscales)	.949^**^	.858^**^	1

^**^Correlation is significant at the 0.01 level (2-tailed)

### The final CFA model

The final model did not achieve non-significance, indicating that the global model did not reproduce the observed covariances among the 11 items very well. However, this is common and likely due to the small sample size and ordinal nature of the factors, which are not parametric. Thus, we also evaluated other measures of model fit, as shown in Table 6. The root mean square error (RMSE) improved with each iteration of the model, with the final model at 0.062, which is considered acceptable (i.e., well below the 0.080 cutoff) and is an improvement over the original model. In addition, the comparative fit index (CFI) improved as the model was refined, and the final value of 0.971 is close to the upper limit of this measure (1.00), indicating good model fit. The standardized root means square residual (SRMR) decreased across model iterations, with the final model below the cutoff of 0.1 (Table 6).

**Table 6:** Model Fit Statistics for COVISS-HCWs

Model Fit Statistics for COVISS-HCWs, (*n* = 350)				
**Model (# of Items)**	**CFI (> 0.9)**	**TLI (> 0.9)**	**RMSEA (< 0.1)**	**SRMR (< 0.1)**
Final Model (11)	0.971	0.961	0.062	0.038
Model #1 (14)	0.948	0.937	0.065	0.0456

Abbreviations: *CFI* comparative-fit index, *RMSEA* root mean square error of approximation, *SRMR* standardized root mean squared residual, *TLI* Tucker-Lewis Index

Table 7 demonstrates that all factors in the final model loaded highly and significantly onto their theoretical constructs, indicating that the survey items are all important indicators of the theoretical constructs (latent factors), as theorized.

**Table 7:** Standardized factor loadings, p-values, 95% Cis, and R2 for survey items in the final model for COVISS-HCWs

The final 11-item Stigma (*n* = 350)		Scoring structure	Mean ± SD; median (range)	Standardized factor loadings (95% CI)		*P*-value	R^2
Item				Factor 1	Factor 2		
Cronbach's Alpha = 0.87							
Item 1	[You were blamed for the nature of your work and spreading the infection]	1–2-3–4-5	2.30 ± 1.15; 2 (4)	0.673 (0.607—0.743)	-	0.005	0.453
Item 2	People seemed uncomfortable dealing with you	1–2-3–4-5	2.11 ± 1; 2 (4)	0.78 (0.709—0.837)	-	0.012	0.608
Item 3	[You felt unwanted]	1–2-3–4-5	1.75 ± 0.93; 1 (4)	0.81 (0.739—0.864)	-	0.013	0.656
Item 4	[You worried about being stereotyped]	1–2-3–4-5	1.84 ± 1; 2 (4)	0.766 (0.72—0.823)	-	0.009	0.587
Item 5	[You have been stared or pointed at in your community]	1–2-3–4-5	2.20 ± 1.108; 2 (4)	0.615 (0.513—0.688)	-	0.009	0.378
Item 6	[You felt that others were scared from you]	1–2-3–4-5	2.31 ± 1.09; 2 (4)	0.736 (0.687—0.788)	-	0.004	0.541
Item 7	[You feared being negatively judged by others]	1–2-3–4-5	1.95 ± 1.07; 2 (4)	0.543 (0.449—0.624)	-	0.009	0.295
Cronbach's Alpha = 0.85							
Item 8	[Your family or friends preferred not to keep their kids around you]	1–2-3–4-5	1.77 ± 1.05; 1 (4)	-	0.831 (0.779—0.873)	0.011	0.691
Item 9	[People avoided eating or drinking with you]	1–2-3–4-5	1.60 ± 0.89; 1 (4)	-	0.835 (0.775—0.889)	0.01	0.698
Item 10	[People kept a very large distance when interacting with you]	1–2-3–4-5	2.11 ± 1.098; 2 (4)	-	0.673 (0.589—0.736)	0.012	0.453
Item 11	[People avoided any physical interaction with you]	1–2-3–4-5	2.04 ± 1.094; 2 (4)	-	0.729 (0.651—0.783)	0.013	0.531

Overall Scale (COVISS-HCWs) (Cronbach’s α = 0.909)

In the Harmfulness and Inferiority Subscale, the survey item that explained the largest proportion of variance (65.6%) was Q3, while for the Avoidance Subscale, Q9 explained the largest proportion of variance (69.8%). Thus, the COVISS-HCWs survey is a reliable and valid measure of Perceived Harmfulness and Inferiority, and Avoidance. The items in these subscales are strongly related to the latent variables (Perceived Harmfulness and Inferiority, and Avoidance) as specified, and can be utilized for assessing the same in HCWs.

Confirmatory factor analysis resulted in reducing the survey from 14 to 11 items (Table 7).

For the final model, convergent validity was explored using average variance extracted (AVE)and composite reliability (CR). A model is generally regarded as having acceptable convergent validity if the AVE is at least 0.50 and the composite reliability CR is above 0.70.

**Fig. 1:**
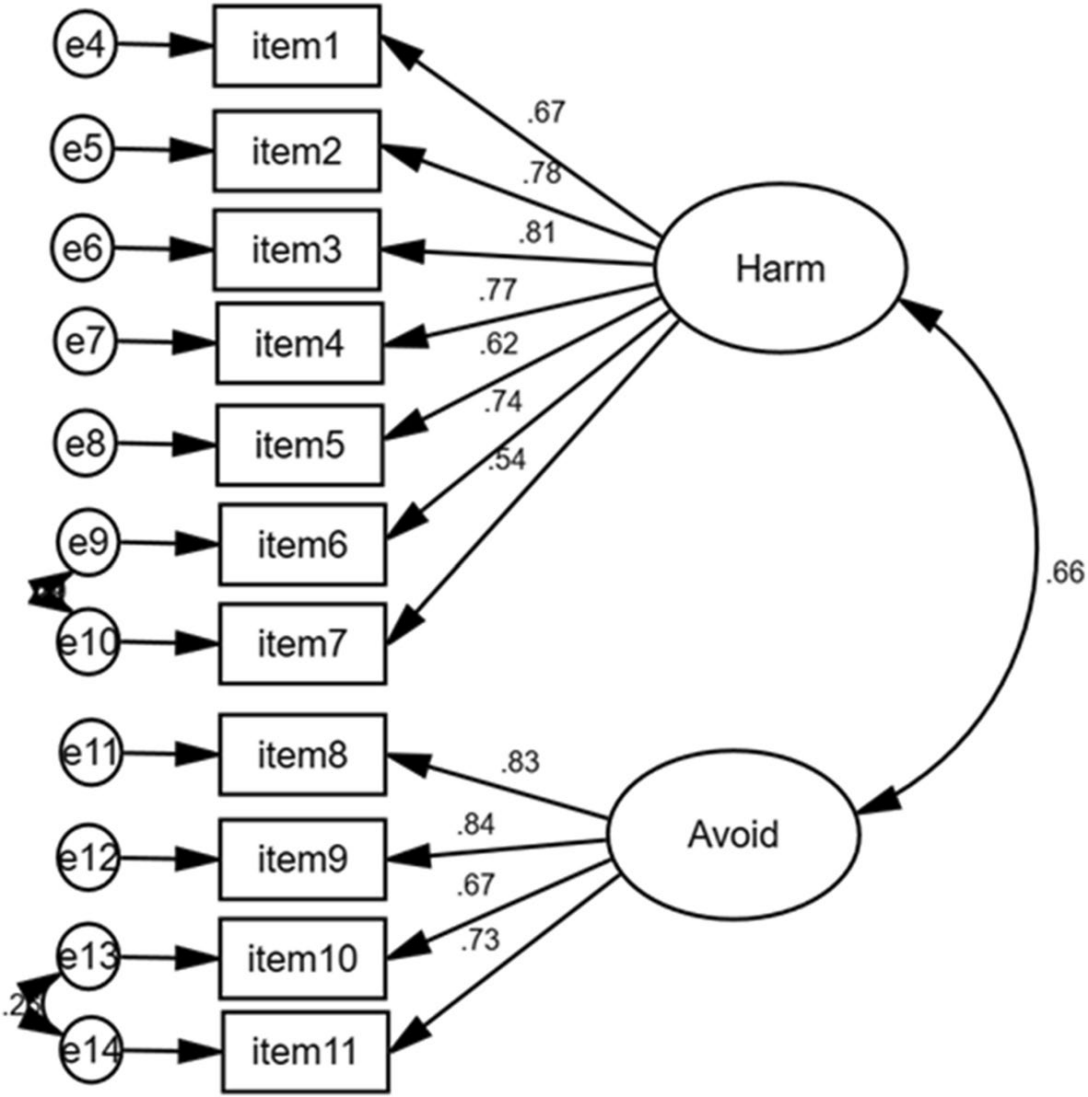
Final CFA Model for COVISS-HCWs

Referring to Table 4, the CR for all constructs is above 0.70 and the AVE values are greater than 0.50. The discriminant validity was assessed using Fornel and Larcker (1971) by comparing the square root of each AVE in the diagonal with the correlation coefficients (off-diagonal) for each construct in the relevant rows and columns. However, the square root of the AVE for Factor was greater than the absolute value of the correlation with another factor. Overall, convergent and discriminant validity can be accepted for this measurement model and the final model is presented in Fig. 1.

### Frequency of stigmatization among healthcare workers

We summarized participants’ responses to each COVISS-HCWs item into no stigma for those who answered with “never” and “rarely”; neutral for “sometimes”; and stigma for “very often” and “always”. Frequency distribution of stigma experience is presented in Table 8. For each subscale and COVISS-HCWs, the mean number of participants and their percentage under each stigma category was calculated. Our findings indicate that 9.86% of the participants reported being stigmatized. Harmfulness and inferiority, and avoidance were experienced by 10.43% and 9.30% of the participating healthcare workers, respectively.

**Table 8:** Descriptive analysis based on stigmatization

As a health care worker fighting against COVID-19 disease, have you ever experienced any of the following situations?	Answers, N (%)		
	**No stigma**	**Neutral**	**Stigma**
Q1. You were blamed for the nature of your work and spreading the infection	426 (60.9%)	157 (22.4%)	117 (16.7%)
Q2. People seemed uncomfortable dealing with you	478 (68.3%)	161 (23%)	61 (8.7%)
Q3. You felt unwanted	571 (81.6%)	92 (13.1%)	37 (5.3%)
Q4. You worried about being stereotyped	534 (76.3%)	116 (16.6%)	50 (7.1%)
Q5. You have been stared or pointed at by people in your community	422 (60.3%)	190 (27.1%)	88 (12.6%)
Q6. You felt that others were scared from you	414 (59.1%)	188 (26.9%)	98 (14%)
Q7. You feared being negatively judged by others	506 (72.3%)	130 (18.6%)	64 (9.1%)
**Feeling of Shyness and Inferiority Subscale**	**479 (68.43%)**	**148 (21.14%)**	**73 (10.43%)**
Q8 Your family or friends preferred not to keep their kids around you	555 (79.3%)	81 (11.6%)	64 (9.1%)
Q9 People avoided eating or drinking with you	594 (84.9%)	72 (10.3%)	34 (4.9%)
Q10 People kept a very large distance when interacting with you	470 (67.1%)	148 (21.1%)	82 (11.7%)
Q11 People avoided touching you	488 (69.7%)	131 (18.7%)	81 (11.6%)
**Avoidance Subscale**	**527 (75.3%)**	**108 (15.4%)**	**65 (9.3%)**
**COVISS-HCWs**	**503 (71.85%)**	**128 (18.29%)**	**69 (9.86%)**

Moreover, we used Chi-square test to evaluate the distribution of responses into demographic variables. The results indicate that age significantly correlated with the presence of COVID-19 stigma (*P* < 0.001), with a higher percentage of participants between the age of 18–29 years reporting stigmatization. Moreover, financial status significantly correlated with experiencing stigma (*P* = 0.013), with a higher percentage of low-income participants feeling stigmatized (32.3%), as opposed to 15.7%, 14.3%, and 12.9% of participants of medium, good, and excellent financial status, respectively, indicating a dose response relationship. No significant association was reported between medical specialty and stigmatization frequency.

Most participants (80.4%) had neighbors, relatives, or co-workers who were aware that they worked in hospitals or clinics, or that they were in contact with Coronavirus-infected individuals (81.5%). And most participants (96.6%) had occasional or close/daily contact with COVID-19 patients. Importantly, the intensity of contact with SARS-CoV2-infected people correlated positively with stigmatization (*P* = 0.004), as 19.4% of those who were in close daily contact compared to 14.6% of those who only had occasional contact felt stigmatized. On the other hand, only 7.5% of those who had no contact with COVID-19 patients reported feeling stigmatized.

### Stigmatization correlates with COVID-19 vaccine attitudes among healthcare workers

We hypothesized that stigmatization increases the likelihood of accepting a vaccine that protects them from COVID-19. The attitudes of healthcare workers towards the upcoming SARS-Cov2 vaccines were investigated; where 25.7% said they would not take it, 40.9% said they would take it despite being fearful of its possible side effects, and 27.1% said they would take the vaccine without fear. The rest of the participants (6.3%) did not respond to this question.

Interestingly, we found that SARS-Cov2 vaccine attitudes significantly correlated with feeling stigmatized (*P* < 0.001). While 69.8% and 73% of those in the “no stigma” and “neutral” categories reported they will take the vaccine, 83.6% of participants in the “stigma” category stated they will take the vaccine. Additionally, among the participants who said they will take the vaccine despite fear of its side effects, 21.9% felt stigmatized, while 14.5% of those who stated they will take the vaccine without fear, and only 10.2% of those who stated they will not take the vaccine felt stigmatized. These data suggest that those who feel stigmatized may find a way out of their situation through vaccination, even if they had fears around a new vaccine. This, however, needs further investigation.

## Discussion

Stigma develops towards diseases in situations where a person is considered to be the cause of disease, incurable or degenerative, when the disease seems to cause adverse effects for others, and when it has visible signs on the patient’s body. These situations are compatible with many diseases such as tuberculosis, leprosy, HIV/AIDS, H1N1 and severe acute respiratory syndrome (SARS). During the SARS outbreak, HCWs were prevented from interacting outside and inside the hospital with their colleagues. Furthermore, habits that require mask removal such as eating, and drinking were done alone or outside the hospital [20]. In addition, HCWs had concerns about their safety, transmitting the disease to family members, stigmatization, and social isolation. On the other hand, the relationship between patients and staff became blurred as they experienced a strong emotional identification with their colleagues who were now infected which increased anxiety regarding their competence and skills [20]. One study reported that HCWs realize that their work is less valued due to the stigma that comes from close dealing with HIV-infected patients [21]. While currently, during the COVID-19 pandemic, HCWs in Mexico and Malawi were found to use bicycles, as they were prevented from using public transportation and were subjected to physical abuse [22]. In India, doctors and nurses dealing with COVID-19 patients faced social stigmatization as they were fired from their rented homes and were even attacked during their duties [22]. Recently, COVID-19 joined the list of diseases that trigger stigmatization. However, this disease has different pathologies and spreads relatively faster than many other diseases, which is likely to cause more panic among community members. As in the case with previous outbreaks, HCWs during COVID-19 are at the frontline confronting the pandemic and may face stigmatization. Experiencing stigma negatively affects HCWsʼ psychological status which may reflect on their medical performance and influences the spread of disease. According to the WHO, HCWs treating COVID-19 are considered as a stigma-vulnerable group during the pandemic along with travelers to infected countries and symptomatic patients [23, 24]. Considering the widespread nature and continuing relevance of COVID-19 and its associated stigma, it is important to develop a COVID-19-specific stigma scale in order to evaluate the prevalence of stigma during the pandemic. A universal, reliable, and valid scale for evaluating presence of COVID-19 stigma among HCWs is still lacking. The main objective of the current study, that included 700 HCWs from five of Damascus’s dedicated COVID-19 hospitals, was to develop the COVID-19 Stigma Scale (COVISS**-**HCWs) in order to investigate the prevalence of COVID-19-associated stigma among HCWs and its possible predictive factors. Accordingly, we developed and divided COVISS-HCWs items into two subscales (harmfulness, and inferiority, and avoidance) depending on EFA. Confirmatory factor analysis (CFA) resulted in reducing COVISS from 14 to 11 items. Importantly, responses to all items and all subscales highly correlated with the overall scale indicating COVISS-HCWs reliability. We found that 69 HCW (9.86% were stigmatized according to our validated score. Few studies reported varying proportions of COVID-19 stigma using non-validated stigma tools among Indonesian, Egyptian, Burkina Faso, Nigerian, and Ethiopian physicians that were 21.9%, 31.2%, 66%, 67%, and 88% respectively [25–27]. Furthermore, Adalberto et al. suggested non- validated scale to determine COVID-19 stigma-discrimination toward HCWs [28]. In general, studies that attempted to use a definite validate COVID-19 stigma scale involved either non-HCWs such as general population and recovered infected patients or a particular group of HCWs except the Vietnamese and Egyptian studies [9, 29–32].The later attempted to adapt the SARS stigma scale to form a valid scale for assessing the presence of covid-19 among physicians [9].

The scale consists of 16-item, each item had four possible responses distributed as 1–4 Likert scale points, allocated into three subscales personalized stigma (8 items); concerns of disclosure and public attitudes (5 items); and negative experiences (3 items) with Cronbach’s α for the three subscales 0.90, 0.66, and 0.78 respectively and 0.90 for overall scale [9]. In this study, we adapted some questions from Nyblade HIV and TB stigma scales to fit Covid 19 HCWs [11, 12].Whereas, the other questions were developed by our team to Covid 19 HCWs. Our final novel questionnaire consists of 11 items only, each item had five possible responses and distributed as 1–5 Likert scale points which is more precise than 1–4 Likert scale points, allocated into two subscales harmfulness and inferiority (7 items); and avoidance (4 items) with Cronbach’s α for the two subscales 0.87 (very good), and 0.85 (very good), respectively and 0.909 for overall scale. The Egyptian study depends on the SARS stigma scale that developed its scale from Berger HIV scale which is developed to assess stigma among infected HIV patients not particularly to assess stigma among HCWs [9, 33, 34]. While we adapted some questions from Nyblade HIV and TB stigma scales that designed their scales to assess stigma among HCWs [11, 12]. Another small study included only 61 Vietnamese HCWs attempted to develop validated scale that administrated in Vietnamese language, while in this study the scale was administrated in English language to increase its utility in the world [32]. However, the published scale was different from the SARS HIV and tuberculosis stigma scale which we used to develop the COVISS-HCWs scale [11, 12, 33]. The first subscale concerns harmfulness and inferiority, and the second subscale was avoidance faced by HCWs due to communication behaviors. Responses to all items and all subscales highly correlated with the overall scale. Further analysis of validity measures allow us to conclude that the final model displayed good convergent but mixed divergent validity [35]. In this study, financial status significantly correlated with experiencing stigma as it appears in 32,3% of low-income participants, which is nearly similar to other studies conducted in low-income countries (37%) [36]. This can be explained that low-income countries are more likely to attend stress and as a result they may have huge psychological pressure on population as well as HCWs. In addition, HCWs who have less contact with COVID-19 patients were less stigmatized than others who had occasional contact, and also less than who had daily close contact with them, 7.5%, 14.6% and 19.4% respectively. This result is similar to other studies which end up with 3.5 times stigmatization when HCWs deal with COVID-19 inpatients [25]. Stigma aggravates stress, post-traumatic stress syndrome, burnout syndrome, inability to concentrate and make decisions, negative self-image and concerns about the public attitude in addition to feeling guilty and avoiding communication with friends and family members [4, 32, 37–39]. As a result, inability to work properly and cope with the various situations was seen in the presence of HCWs stigmatization and mental health damage [40, 41]. Our findings show that HCWs are being stigmatized and need to have help from health policy makers and officials. Moreover, it is essential to provide effective psychiatric, physical, ethical and social support from their families, friends and health professionals after quarantine or hospitalization to get over the negative consequences. This may reduce the long-term mental effects of the disease [42].

## Study strengths and limitations

This is the first validated scale to evaluate COVID19 stigma rates among health care workers using only 11-items. The scale was adapted from others that were specifically developed and validated for HCWs, with additional questions that we tailored to fit the COVID19 context. However, our study is cross-sectional, hence it demonstrated associations but not causal relations between the studied elements. It was also based on a self-reported questionnaire, not direct observation. Therefore, responder bias cannot be excluded, and findings may be biased by social desirability to prove HCWs distress during the pandemic. Moreover, resident doctors represented the vast majority of our specimen which may represent a specific group of HCWs, and thus may limit the generalizability of our findings to all HCWs.

## Conclusion

Stigma towards HCWs in COVID-19 pandemic is a widespread phenomenon in countries all over the world. It should be identified with its categories harmfulness and inferiority, and avoidance. 9.86% of HCWs were stigmatized. COVISS-HCWs demonstrated very good internal consistency and construct validity among this sample of Syrian's HCWs. These satisfactory properties make the COVISS-HCWs suitable for utilize by health care providers.

## Data Availability

The datasets generated during and analyzed during the current study are not publicly available due to containing information that could compromise the privacy of research participants but are available from the corresponding author on reasonable request.
